# Use of an Emergency Manual During an Intraoperative Pulmonary Arterial Rupture, Hypoxemia, and Bradycardia

**DOI:** 10.7759/cureus.6838

**Published:** 2020-02-01

**Authors:** Jeffrey Huang, Xiaofeng Huang, Yin Guan, Xiaobin Wang, Shovna Mishra

**Affiliations:** 1 Anesthesiology, University of Central Florida College of Medicine, Orlando, USA; 2 Anesthesiology, Gansu Provincial Cancer Hospital, Lanzhou, CHN; 3 Anesthesiology, Lanzhou University Second Hospital, Lanzhou, CHN; 4 Anesthesiology, Oak Hill Hospital, Brooksville, USA

**Keywords:** emergency manual, crisis, video-assisted thoracic surgery (vats), hypoxemia, hemorrhage

## Abstract

The use of an emergency manual can improve team performance on critical steps during crisis events. Measures of improved performance have so far been captured through survey and simulation data; however, real-life case studies showing successful use of the manuals are fewer in number. The case of a patient with an unexpected rupture of the pulmonary artery, hypoxemia, and bradycardia during a video-assisted thoracic surgery lobectomy is described here. Relevant sections of Stanford University Operating Room Emergency Manuals were activated immediately and used during the rescue. The team of surgeons, anesthesiologists, and nurses managed the crisis in an orderly, smooth, and efficient manner, and the patient recovered without any complication. The use of the emergency manual reinforced by regular simulation-based training benefited the team and ultimately, the patient’s safety.

## Introduction

Video-assisted thoracic surgery (VATS) lobectomy is a procedure with proven benefits over open lobectomy and is currently considered the preferred approach. However, bleeding related to vascular injury is still a very troublesome, although not common, complication during this procedure. Nonetheless, intraoperative hemorrhage can cause patient death. The operating room (OR) team has to learn and practice how to manage this critical situation efficiently using an emergency manual (EM).

EM, which can be defined as a cognitive aid, checklist, or tool to guide decision making of the anesthesia care team and OR personnel, is synergistic with simulation-based training in enhancing team performance [1]. The purpose of the EM is to reduce the reliance on memory, which is prone to human error, especially in situations where information that may not be used on an everyday basis, such as in emergencies [2]. Harvard studies showed four to six times less adherence to emergency protocol without EM as opposed to when EM were used [2,3]. At Virginia Mason Medical Center, it was found that EM increased knowledge retention of protocol (p=.031), doubled the number of tasks completed as part of the protocol, and even greater adherence was found when a dedicated reader was assigned to the team [2,4].

EM has undergone phases of adoption-acquire, familiarize, clinically use, and finally integration into daily practice-in China [2]. Three cognitive aids including the Stanford University Operating Room Emergency Manual, Ariadne Lab Operating Room Crisis Checklists, and Society for Pediatric Anesthesia Crisis Checklists were translated to Chinese and introduced to Chinese hospitals in 2015. The implementation of EM was promoted through online forums, conferences, simulation-based training, and competitions. This significantly increased the awareness of Chinese anesthesiologists about crisis in the OR and the willingness to use EM [5,6].

During simulation trainings and drills, the situations, environment, and equipment were as realistic as possible. Team members took turns to play different roles. Mobile phones were used to record the exercise and then debrief and analyze immediately after each exercise, so every member knew how to improve. The repeat exercise not only improved communication, collaboration, and decision-making capabilities but also developed the team’s awareness of the acute events and ability to apply the EM. The effects of integrating EM paired with simulation training can be seen in the following case presentation.

## Case presentation

An 81-year-old Chinese male (height 165 cm, weight 74 kg), former smoker, was found to have left upper lobe lung cancer during a routine health examination. His past medical history included essential hypertension for 10 years, controlled with nifedipine, and coronary heart disease. The patient’s vitals were within normal limits. Airway examination was normal with Mallampati grade II. Systemic examination revealed normal cardiorespiratory findings. Preoperative labs showed hemoglobin (Hb) 16.5 g/dL, hematocrit (Hct) 50.6%, and platelets 136 × 109/L. Coagulation tests normal. Electrocardiogram was within normal limits. Cardiac echography reported an ejection fraction of 66%. The patient was categorized as American Society of Anesthesiologist grade III, and general anesthesia with invasive arterial pressure monitoring was planned.

General anesthesia was induced with the patient in the supine position. He was intubated with a left-sided 35 Fr double-lumen endotracheal tube (ETT) to obtain one-lung ventilation. The left radial artery line and right internal jugular double-lumen catheter were placed. The patient remained hemodynamically stable (blood pressure [BP] 120/70 mmHg, heart rate [HR] 80 bpm, SpO_2_ 94%) until the time of the dissection of left lung apicoposterior segmental artery. The left pulmonary artery was accidentally torn, and heavy bleeding was noticed immediately. The patient’s vital signs changed immediately (BP 84/52 mmHg, HR 121 bpm, SpO_2_ 94%). There was approximately 2,000 mL of blood in the suction canister within five minutes.

The anesthesiologist immediately reduced the inhalation agent concentration, started pressurized infusion, and activated the emergency protocol for a crisis event in the OR. As in simulation training, the anesthesiologist assumed the role of the leader. He asked the circulating nurse to call for help and to retrieve the blood stored for emergency and the code cart. The additional anesthesia providers arrived immediately. An anesthesiologist was assigned the role of performing anesthesia procedures if necessary. Another team member was designated as the reader and was asked to read aloud the Hemorrhage section of the Stanford University Operating Room Emergency Manuals. The recommendations in the Hemorrhage section were reviewed one by one and followed if appropriate, which included high flow 100% O_2_, rapid infusion, changing position, using vasopressors, sending a type and cross sample, maintaining normothermia, and monitoring for hypocalcemia.

Communication was maintained with the surgical team, as the surgeons decided to convert from VATS to open surgery and called for additional help from the senior surgeon. The patient’s BP decreased to 62/31 mmHg, HR increased to 132 bpm, and SpO_2_ was 89%. Lab tests showed Hb 8.6 g/dL and Hct 26.4%. 100% high flow oxygen, rapid infusion, norepinephrine drip, and multiple epinephrine boluses were used to maintain BP. Massive blood transfusion protocol was initiated. Seven units of packed red blood cells (PRBCs) and 700 mL of fresh frozen plasma (FFP) arrived in the OR. After transfusion, the patient’s BP increased to 93/52 mmHg and HR decreased to 113 bpm. 

The surgical team started to repair the vessel. However, the patient’s oxygen saturation fell precipitously. The patient became hypoxic, bradycardic, and hypotensive. The leader asked the reader to read aloud the Hypoxemia section of the Stanford University Operating Room Emergency Manuals. Each step was checked and executed, fiber optic bronchoscopy was performed, and it was found that the ETT was dislodged. The ETT was repositioned. SpO_2_ gradually increased from 37% to 93%, HR increased from 45 to 89 bpm, and BP increased from 67/40 to 110/75 mmHg. The patient’s vital signs remained stable. The estimated blood loss was 3,200 mL. Total fluid and blood given during surgery were lactated Ringer's solution 4,500 mL, hydroxyethylstarch 1,500 mL, normal saline 300 mL, PRBCs seven units, and FFP 700 mL. Cryoprecipitate five units, albumin 20 g, and calcium gluconate 1 g were given at the end of the case. He was transported to ICU with stable condition. The rest of hospital course was uneventful.

## Discussion

There are many advantages associated with VATS lobectomy over open lobectomy, such as shorter length of stay, fewer perioperative complications, and greater likelihood of disposition of independent home discharge [7]. However, the procedure is not without risks as the intraoperative conversion rate to open surgery ranges from 3% up to 23% for various reasons, with the most frequent reason being vascular injury [8-10]. In a study of reasons for conversion to open lobectomy during VATS lobectomy in 40 cases, there were 13 cases in which vascular injury was the cause for conversion. Approximately 77% of the cases were upper lobectomies, indicating a higher occurrence of vascular injury during upper lobectomy [10]. Although the overall incidence of catastrophic complications during VATS lobectomy, such as injury of the pulmonary artery, pulmonary vein, bronchi, abdominal organs, and injury during mediastinal nodal dissection, is low, there are no predicting factors of such events. Not even level of experience in the surgeon was found to be a factor [11]. Therefore, anesthesiologists should always be vigilant and prepare for the potential catastrophic complications through use of cognitive aids like EM.

The dislodgement of a double-lumen tube (DLT) caused by the surgeon’s operation is not very uncommon. DLT displacement usually occurs when surgery is difficult and involving traction at the hilum [12]. Any attempt to ventilate the patient during the tube’s relocation to its correct position will almost certainly result in expansion of the operated lung; this in itself can create a clinical emergency. If the displacement were to occur at a time when surgical exposure is vital to repairing pulmonary vessels, as in this case, hypoxemia may hinder the surgeon’s ability to control the hemorrhage. Under normal circumstances, the anesthesiologist could often identify and fix it quickly. However, at the time, the stress related to the hemorrhage, hypotension, and rapidly decreasing SpO_2_ may have caused a delay in recognition of the dislodged tube. The team members were exhausted, experiencing hemorrhage immediately followed by hypoxemia. Fortunately, the anesthesiologist determinedly decided to use the EM, and the situation improved quickly.

The EM as a cognitive aid can reduce the missing of key steps in times of crisis events [13]. Implementing EM had been shown to relieve stress and improve efficiency and teamwork [2,13-15]. However, due to the difficulty of measuring the behaviors which may enhance patient safety, including leadership and role assignment, enhanced communication, situational awareness, and teamwork, most published articles about OR EM and patient safety are based on simulation study and survey [2,16]. Reporting the successful usage of the EM in real cases as in this one, where hemorrhage and hypoxemia in VATS lobectomy were plausible but unlikely to occur, offers insight on ideal application and integration of EM. 

## Conclusions

The effective application of EM during these two acute events, hemorrhage and hypoxemia, resulted from the repeat simulation trainings and drills. The members of the rescue team were quick to assume roles, familiar with the content and format of the manual, able to complete required tasks outlined, and communicated effectively. In short, as a supplement to clinical knowledge, EM combined with repeat simulation exercises improved the team’s ability to manage real-life crisis events in the OR as demonstrated in this case. EM increased the confidence of the team to handle acute events in the future. Most importantly, the EM training regimen saved the patient’s life, allowing a smooth recovery from the unexpected crises.
